# Association of Short-Term Exposure to PM_2.5_ with Blood Lipids and the Modification Effects of Insulin Resistance: A Panel Study in Wuhan

**DOI:** 10.3390/toxics10110663

**Published:** 2022-11-04

**Authors:** Jinhui Sun, Shouxin Peng, Zhaoyuan Li, Feifei Liu, Chuangxin Wu, Yuanan Lu, Hao Xiang

**Affiliations:** 1 Department of Global Health, School of Public Health, Wuhan University, 115# Donghu Road, Wuhan 430071, China; 2 Global Health Institute, Wuhan University, 115# Donghu Road, Wuhan 430071, China; 3 Environmental Health Laboratory, Department of Public Health Sciences, University of Hawaii at Manoa, 1960 East West Rd., Biomed Bldg D105, Honolulu, HI 96822, USA

**Keywords:** panel study, blood lipids, PM_2.5_, short-term exposure, HOMA-IR

## Abstract

Results of previous studies about the acute effects of fine particulate matter (PM_2.5_) on blood lipids were inconsistent. This study aimed to quantify the short-term effects of PM_2.5_ on blood lipids and estimate the modifying role of insulin resistance, reflected by the homeostasis model assessment of insulin resistance (HOMA-IR). From September 2019 to January 2020, the study recruited 70 healthy adults from Wuhan University for a total of eight repeated data collections. At each visit, three consecutive days were monitored for personal exposure to PM_2.5_, and then a physical examination was carried out on the fourth day. The linear mixed-effect models were operated to investigate the impact of PM_2.5_ over diverse exposure windows on blood lipids. With the median of the HOMA-IR 1.820 as the cut-off point, participants were assigned to two groups for the interaction analyses. We found the overall mean level (standard deviation, SD) of PM_2.5_ was 38.34 (18.33) μg/m^3^. Additionally, with a 10 μg/m^3^ rise in PM_2.5_, the corresponding largest responses in triglyceride (TG), total cholesterol (TC), low-density lipoprotein cholesterol (LDL-C), as well as high-density lipoprotein cholesterol (HDL-C), were −0.91% (95% confidence interval (CI): −1.63%, −0.18%), −0.33% (95% CI: −0.64%, −0.01%,), −0.94% (95% CI: −1.53%, −0.35%), and 0.67% (95% CI: 0.32%, 1.02%), respectively. The interaction analyses revealed that a significantly greater reduction in the four lipids corresponded to PM_2.5_ exposure when in the group with the lower HOMA-IR (<1.820). In conclusion, short-term PM_2.5_ exposure over specific time windows among healthy adults was associated with reduced TG, TC, as well as LDL-C levels, and elevated HDL-C. Additionally, the association of PM_2.5_–lipids may be modulated by insulin resistance.

## 1. Introduction

Plenty of countries are suffering from poor air quality and related health problems. The Global Burden of Disease Study 2019 (GBD 2019) stated that air pollutants accounted for a total of 6.67 million deaths worldwide, and the percentage of attributable disability adjusted life year (DALYs) of particulate matter had increased from 2.7% in 1990 to 4.7% in 2019 [1]. It is currently believed that ambient air pollutants probably play a significant part in dyslipidemia development [2,3], a chronic disease which many risk factors can contribute to [4,5]. According to the GBD 2019, more than four million deaths worldwide in 2019 could be attributed to high LDL cholesterol [1]. Known as dyslipidemia, it is attributed to abnormally higher levels of triglycerides (TG), total cholesterol (TC), low-density lipoprotein cholesterol (LDL-C), or lower high-density lipoprotein cholesterol (HDL-C) [6]. Within the development and progression of atherosclerosis and cardiovascular disease, dyslipidemia has been considered an important modifiable risk factor [7,8,9]. In addition, a great many observations have reported that abnormal lipid levels were related to cardiovascular disease morbidity and mortality, overall mortality, and the prevalence of ischemic stroke [10,11,12].

There have been numerous investigations into the relationship between long-term fine particulate matter (PM_2.5_) and blood lipids, and the findings are all relatively consistent. In particular, a positive association of PM_2.5_ with TG, TC, as well as LDL-C, and a negative association with HDL-C were proposed in those studies [3,13,14,15]. For example, a rise of 10 μg/m^3^ in PM_2.5_ was accompanied by a 2.23% (95% confidence interval (CI): 1.44, 3.02), 0.92% (0.64, 1.20), and 3.04% (2.61, 3.47) increase in TG, TC, and LDL-C levels, respectively, as well as a 2.03% (−1.69, −2.37) decrease in HDL-C levels [13]. However, the current findings on the acute effects of ambient particulate matter on blood lipids are varied. For example, a study based on patients with type 2 diabetes found that each 10-μg/m^3^ rise in PM_10_ was accompanied by changes of 0.45% (0.08, 0.82), 0.83% (0.21, 1.45), and 0.29% (0.10, 0.49) in TC, LDL-C, and HDL-C, respectively, and −0.01% (−1.01, 1.00) in TG [2]. One US study that used the mixed-effects models interestingly indicated a positive association between a 30-day particle number and HDL-C, and the association was stronger in those people who had higher HDL-C levels [16,17]. A panel study in North Carolina reported that, when PM_2.5_ increased by 1 μg/m^3^, the TG and TC changed by −0.63 (95% CI: −2.29, −1.02) and −0.06 (−0.49, 0.36), while the statistics did not show any significant differences [17]. The results of one cohort study suggested that short-term PM_2.5_ exposure (per interquartile range (IQR) for lag 1 day) and TG (−0.3%; 95%CI: −1.1, 0.5), TC (−0.1%; −0.4, 0.2), LDL-C (0.03, −0.4, 0.5), and HDL-C (−0.01, −0.3, 0.3) did not have any statistically significant association [18].

Previous research has linked PM_2.5_ to elevated blood glucose [19]. Likewise, studies have shown that hyperglycemia and dyslipidemia are closely related [20,21]. In addition to being components of metabolic syndrome, they are both contributors to atherosclerosis [22,23]. Furthermore, recent research concluded that the adverse effects of chronic air pollutants exposure on lipids could be strengthened by high levels of blood glucose among healthy adults [24]. However, no studies have yet explored what role blood glucose might play in the acute association of fine particles with lipids. Therefore, considering the link between air pollutants and blood glucose, as well as the connection between blood glucose and blood lipids, we hypothesized that glucose homeostasis would influence the short period impact of PM_2.5_ exposure on lipid profiles. In this study, the homeostasis model assessment of insulin resistance (HOMA-IR) was used as an indicator of insulin resistance [25,26].

On the whole, though extensive studies have been conducted investigating the impact of particulate matter on blood lipids over the past few decades, the conclusions about the acute effects are not consistent, which indicates that the mechanism of the short-term PM_2.5_–lipids association is complex. Additionally, there is a paucity of attention paid to the role of glucose homeostasis in the PM_2.5_–lipids association. Thus, to examine the acute effects of PM_2.5_ on blood lipids, as well as the modifying role of the HOMA-IR, we designed this panel study.

## 2. Materials and Methods

### 2.1. Participants and Study Design

From September 2019 to January 2020, the panel study was conducted in Wuhan University. The participants were limited to healthy students aged 18–30 who live within 1 km of the School of Medicine, Wuhan University, they have lived in the current residence for more than two years, and have no plans to leave during the study period. The exclusion criteria for students were: (i) smoked or with a history of alcohol abuse; (ii) clinical diagnosis of chronic diseases such as cardiopulmonary disease; (iii) diagnosed with infectious diseases or used anti-inflammatory drugs, antibiotics, or other drugs in the past one month.

A total of 70 healthy students at Wuhan University were ultimately recruited. Our previous publication has described the visit schedule of the study [27]. In brief, each participant was recruited to complete a baseline questionnaire to collect demographic information (i.e., age, gender, weight, height, etc.). Due to the limitation of the number of instruments, participants were divided into two groups to complete eight clinical visits from 9 September 2019 to 7 January 2020 and the interval between visits was controlled at 1 to 2 weeks. At each clinical visit, the blood lipid levels of each participant were measured. Considering the lagging effect of particulate matter on health, hourly personal PM_2.5_ exposure was monitored for 3 consecutive days prior to lipids measurement. Then, the exposure and the physical examination data of the second group were collected in the same way. Participants were additionally invited to complete a questionnaire on recent physical exercise and dietary intake (i.e., alcohol, caffeine, foods).

Among the 70 subjects, 25 completed all 8 visits, 40 completed 6 or 7 visits, and the remaining 5 subjects completed 4 or 5 visits. In total, we included 480 valid person visits in the subsequent exploration. The Medical Ethics Committee of Wuhan University approved the protocol of this study. Each subject signed an informed consent form prior to the visits.

### 2.2. PM_2.5_ Exposure Measurement

The individual monitor for PM_2.5_ (Ai100, Huawei, Shenzhen, China) was used not only to continuously monitor the hourly concentration of individual exposure to PM_2.5_ but also to measure the ambient temperature and relative humidity. The monitor was equipped with a highly sensitive laser sensor to monitor ambient PM_2.5_ concentrations (ranging from 0 to 1000 μg/m^3^; Resolution: 1 μg/m^3^). The monitors used in our study were calibrated with a standard device, the TSI monitor (Dusttrak 8534, TSI, Shoreview, MN, USA), to evaluate the accuracy. As a result of the analysis for linear regression, the R square value (R^2^) was 0.94, indicating that the individual PM_2.5_ monitor had high accuracy. Participants were required to wear the individual monitors during the 72-h exposure period before each clinical visit to collect the hourly exposure of PM_2.5_. Before the visit was conducted, all participants were trained in the operation of the monitor. The monitors were to be carried during outdoor activities and placed nearby during indoor activities.

### 2.3. Biomarkers Measurement

During each clinical visit, two different tubes were used to collect a total of 10 mL of fasting venous blood by professional personnel from every participant and the fasting blood needed to be collected by 8 a.m. The first 5 mL blood sample was collected with an EDTA-K2 anticoagulant tube and centrifuged after 5–6 shakes. Additionally, a non-anticoagulant tube was used to collect another 5 mL sample and kept still for 30 min before centrifugation. All the blood samples were centrifuged at a speed of 2500 r/min for 15 min. After centrifugation, the plasma or serum specimens were transferred into an Eppendorf tube and stored in a refrigerator at −80 °C. After excluding non-fasting blood and unqualified samples, the remaining 480 blood samples were tested for insulin, glucose, C-reactive protein (CRP), and lipids. The levels of fasting plasma glucose, serum CRP, serum TG, TC, LDL-C, and HDL-C were measured by an automated biochemical analysis instrument (Cobas c701, Roche, Tokyo, Japan). Additionally, an automated immunoanalyzer (Cobas e801, Roche, Tokyo, Japan) was used to measure the concentration of fasting serum insulin.

The HOMA-IR was calculated by taking blood insulin and glucose into account (HOMA-IR = [fasting plasma glucose, mmol/L × fasting serum insulin, μU/mL]/22.5) [28].

### 2.4. Statistical Analyses

Continuous variables were described by means ± standard deviation (SD), categorical variables were described by numbers and percentage (%), and blood lipids were particularly described as quartile (median, 25th percentile (Q1), and 75th percentile (Q3)). Given the non-independence for repeated measurement data, the associations between PM_2.5_ (per 10 μg/m^3^ increase) and blood lipid levels (TG, TC, LDL-C, HDL-C) were evaluated with a linear mixed-effect model (LME). The model allows each subject to act as his or her own control over time and adjusts for between-subject covariates that do not vary over time [29,30]. The fixed and random effects of the LME model were estimated with the restricted maximum-likelihood method [31]. Since the distributions of blood lipid levels were right-skewed, we performed logarithmic transformation for the concentrations of the four blood lipids in the model [32]. According to previous findings, there was a time-lag effect of air pollutants on lipid profiles [2,14,17]. To fully capture the time-lag patterns in the effects of PM_2.5_, we fitted the models by dividing the 72-h concentration of PM_2.5_ into lag time windows (lag 8–16 h, 16–24 h, 24–32 h, 32–40 h, 40–48 h, 48–56 h, 56–64 h, 64–72 h) and moving average time windows (MA 0–8 h, 0–16 h, 0–24 h, 0–32 h, 0–40 h, 0–48 h, 0–56 h, 0–64 h, 0–72 h) [33,34,35]. The time of zero served as the time when the participant was visited. For example, lag 8–16 h referred to the average PM_2.5_ concentration from the 8th hour to the 16th hour prior to each visit; MA 0–24 h referred to the average PM_2.5_ concentration 24 h prior to each visit. Thus, the dependent variables were the levels of log-transformed blood lipids, and the fixed-effect independent variables were the lag and MA concentrations of PM_2.5_. An identification number (ID) was also introduced for each subject as a random-effects intercept to account for autocorrelations due to multiple repeated measurements [34]. In the crude model, only PM_2.5_ concentrations and lipids were included. In the adjusted main model, we additionally included the following covariates: ambient temperature (°C), relative humidity (%), age (years), gender, BMI (kg/m^2^), the day of the week (weekend vs. weekday), exercise status (strenuous exercise vs. no-strenuous exercise), alcohol (drinking vs. no drinking), and caffeine (coffee consumed vs. no coffee). The formula of the LME model was as follows [36]:

*Y_it_* = *β*_0_ + *μ_i_* + *β*_1_
*X*_1*it*_ + *β*_2_
*X*_2*it*_ + … + *β_p_*
*X_pit_* + *β*_PM_2.5__ PM_2.5_ + *ε_it_*

where *i* and *t* denoted the participant and visit time; *Y_it_* was the blood lipid level; *β*_0_ was the intercept for the population mean; *μ_i_* represented the subject-specific random intercept; *β*_1_ *X*_1*it*_ to *β_p_ X_pit_* indicated the potential confounding variables mentioned above; *β*_PM_2.5__ PM_2.5_ was PM_2.5_ concentration over time window; *ε_it_* was the within-subject error term. The effect estimates were back-transformed from a logarithmic scale with the equation percentage change = 100 × [exp (*β*) − 1] [3,13], where *β* was the above-mentioned *β*_PM_2.5__. The associations were presented as percentage changes and 95% confidence intervals (CI) in lipids per 10 μg/m^3^ increments in PM_2.5_, where the percentages were relative to the mean of the targeted lipids [3,37].

Additionally, participants with a HOMA-IR higher than the median of 1.820 were arranged in the higher HOMA-IR group while those lower than 1.820 were in the lower HOMA-IR group [35]. Interaction analyses were then utilized to evaluate the modifications of the stratified HOMA-IR over the PM_2.5_–lipids association. Specifically, in addition to the lower-order terms in the main model described above, we used LME models to assess the modifying effects of insulin resistance by simulating the product interaction terms between different time windows of PM_2.5_ exposure and dichotomous terms of the HOMA-IR (higher vs. lower), respectively. The *p*-value indicated the results of the test for differences in the association of product terms between each group [35]. Additionally, a *p*-value < 0.05 was considered to denote an interaction.

Only the lag time windows (lag 8–16 h, lag 16–24 h, lag 24–32 h, lag 32–40 h, lag 40–48 h, lag 48–56 h, lag 56–64 h, lag 64–72 h) were selected for sensitivity analyses to examine the robustness of the main model. We still used the LME model for statistical analysis and added the adjustments mentioned above into the model. The three models were conducted as follows: Model 1 merely included the participants who completed more than 5 clinical visits; Model 2 excluded those with a CRP of more than 10 mg/L, as the previous study has shown that a CRP greater than 10 mg/L indicates a recent infection [38]; Model 3 further adjusted for the frequency of the recent consumption of fat, fish, egg, and fried food (≥2 times/day; 1 time/day; 2–6 times/week; 1 time/week; Little or never). In total, 456, 473, and 480 person visits were included for Model 1, Model 2, and Model 3, respectively.

The statistical analyses of the study were conducted in R software version 4.0.2 (R Development Core Team, 2020) with the package of “lmerTest”. Statistical significance was determined by a two-sided *p* value < 0.05 in the study.

## 3. Results

### 3.1. Descriptive Statistics

Table 1 described the demographic characteristics among the 70 students. The average age of the study population was 20.37 ± 1.59 years with 80% female. The mean level of BMI was 21.50 ± 2.75 kg/m^2^. The median values for TG, TC, LDL-C, as well as HDL-C, were 0.90, 4.57, 1.98, and 1.38 mmol/L, respectively.

**Figure 1:**
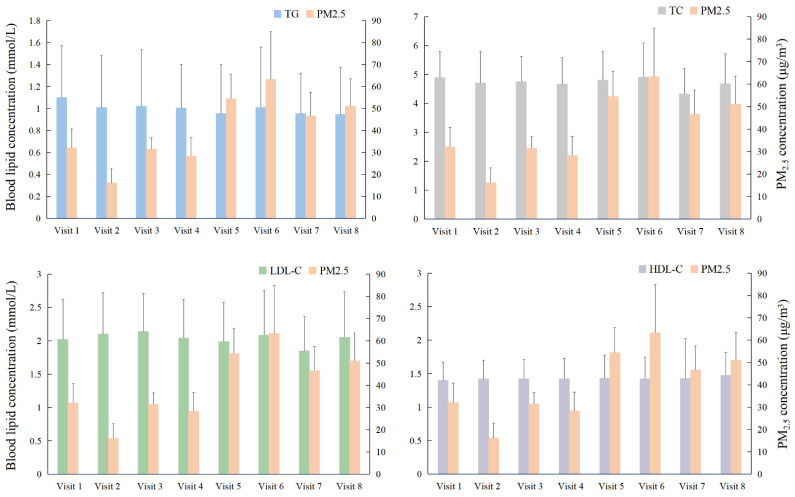
The average TG levels and the 72-h mean concentrations of PM_2.5_ with the corresponding standard deviation in the whole study period. The small “tentacles” at the top of the straight bars indicate the corresponding standard deviation. Abbreviations: PM_2.5_, particulate matter with aerodynamic diameter < 2.5 μm.

The bar charts with errors (mean and standard deviation) for the average lipid levels and the 72-h mean concentrations of PM_2.5_ during the eight visits were presented in Figure 1. Tables S1 and S2 showed the summary of statistics of PM_2.5_ and blood lipids concentrations. There was an average concentration of 38.34 ± 18.33 μg/m^3^ in PM_2.5_ during the study period, and the hourly exposures ranged from 7.38 μg/m^3^ to 115.38 μg/m^3^. The 72-h mean (SD) concentrations of PM_2.5_ from the first visit to the eighth visit were 32.02 (8.74), 16.04 (6.72), 31.45 (5.22), 28.32 (8.47), 54.45 (11.15), 63.25 (21.71), 46.60 (10.84), and 51.08 (12.46) μg/m^3^, respectively. The mean levels (SDs) of TG, TC, LDL-C, and HDL-C during the whole study period were 1.00 (0.46), 4.72 (0.98), 2.04 (0.60), and 1.42 (0.34), respectively.

### 3.2. Association between PM_2.5_ and Blood Lipids

The crude and adjusted associations between PM_2.5_ and blood lipids were shown in Table 2. The crude models suggested that significantly decreased TC and LDL-C, as well as increased HDL-C, was related to individual PM_2.5_ exposure in several windows. For each 10 μg/m^3^ increase in the concentration of PM_2.5_ over MA 0–72 h (i.e., 3-day average), the percentage changes (95% CI) in TG, TC, LDL-C, and HDL-C were −1.06 (95% CI: −2.15, 0.04), −0.05 (−0.52, 0.43), −0.93 (−1.56, −0.30), and 0.30 (−0.23, 0.83), respectively. The largest significant PM_2.5_ effects on TG appeared in MA 0–56 h, which showed that, as the PM_2.5_ increased per each 10-μg/m^3^, the concentration of TG changed by −1.25% (95% CI: −2.29%, −0.20%). The significant positive effect of PM_2.5_ on HDL-C appears at lagged 56–64 h (0.57%; 95% CI: 0.16%, 0.98%) and lagged 64–72 h (0.67%; 95% CI: 0.33%, 1.01%).

After taking the confounding variables mentioned above into account, the adjusted model showed the direction of the significant effect that PM_2.5_ has on TG, LDL-C, and HDL-C remained unchanged, whereas on TC it became negative (Table 2). Specifically, in the adjusted model, for each 10 μg/m^3^ increment in PM_2.5_ over MA 0–72 h (i.e., 3-day average), the percentage changes (95% CI) in TG, TC, LDL-C, and HDL-C were −0.54 (95% CI: −1.67, 0.60), 0.06 (−0.43, 0.55), −0.84 (−1.49, −0.18), and 0.26 (−0.30, 0.81), respectively. For each 10 μg/m^3^ increment in PM_2.5_ over MA 0–24 h (i.e., 1-day lagged), the corresponding changes in blood lipids were −0.34 (−1.36, 0.69), 0.24 (−0.20, 0.69), −0.58 (−1.16, 0.01), and 0.15 (−0.34, 0.65), respectively. For TG, the significant change of −0.91% (95%CI: −0.18, −1.63) appeared in lagged 24–32 h. As for LDL-C, the significant effects were ranged from the minimal −0.66% (−1.20%, −0.12%) to the maximal −0.94% (−1.53%, −0.35%).

### 3.3. Interaction of PM_2.5_ with HOMA-IR and Sensitivity Analyses

**Figure 2:**
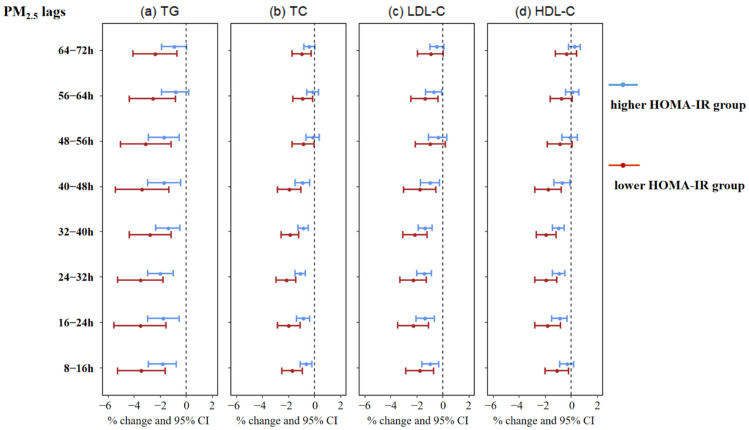
The interaction effects of PM_2.5_ exposure and HOMA-IR on TG (**a**), TC (**b**), LDL-C (**c**), and HDL-C (**d**). X-axis, lagged time window of PM_2.5_ exposure; Y-axis, the estimate effects (percentage change and 95% CI) of PM_2.5_ on blood lipids after stratification by the median of HOMA-IR. Adjusted for temperature, humidity, age, gender, BMI, the day of the week, exercise status, alcohol, and caffeine. Abbreviations: HOMA-IR, homeostasis model assessment of insulin resistance.

As a result of the interaction analyses, PM_2.5_ had a greater effect on the four lipids among the lower HOMA-IR group at several lagged time windows, Figure 2. For instance, in the lower HOMA-IR group, the percentage changes of TG (−3.52%; 95% CI: −5.53%, −1.52%), TC (−1.98%; −2.84%, −1.11%), LDL-C (−2.27%; −3.44%, −1.09%), and HDL-C (−1.80%; −2.78%, −0.81%) were significantly associated with the increment of PM_2.5_ (10 μg/m^3^) with a lag of 16–24 h. However, in the corresponding higher group, the variations of the four lipids were −1.76% (−2.96%, −0.54%), 0.87% (−1.39%, −0.35%), −1.35% (−2.05%, −0.64%), and −0.89% (−1.48%, −0.30%), respectively, and the *p*-value for interaction was less than 0.05 (Table S3). Table S4 showed the results for the sensitivity analysis, which were highly consistent with the adjusted model results in Table 2, indicating good agreement between the models.

## 4. Discussion

Our findings revealed that, among healthy adults, in terms of maximal changes in the four lipids, PM_2.5_ exposure was positively associated with HDL-C levels at PM_2.5_ lag 64–72 h and negatively associated with TG, TC, and LDL-C at PM_2.5_ lag 24–32 h, lag 24–32 h, and MA 0–72 h, respectively. Additionally, it was revealed that PM_2.5_ exposure had greater effects on the four lipid profiles in the lower HOMA-IR group. Since few studies focused on the interaction between glucose homeostasis or insulin resistance and ambient air pollution, our results added epidemiological evidence for the interaction between fine particulate matter and insulin resistance on blood lipids.

It was shown that PM_2.5_ had a generally positive impact on HDL-C levels and a negative impact on the concentration of TG, TC, as well as LDL-C, at specific exposure time windows in this study. For each 10 μg/m^3^ increment of PM_2.5_ exposure, the percentage changes (95% CI) of HDL-C, TG, TC, as well as LDL-C, levels were 0.67% (0.32%, 1.02%), −0.91% (−1.63%, −0.18%), −0.33% (−0.64%, −0.01%), and −0.94% (−1.53%, −0.35%), respectively (according to the significant maximum effect in all exposure time windows for each lipid). Another study in 16 healthy adults aged 18–25 in Provo, Utah, USA, also suggested a positive association between PM_2.5_ and HDL-C. They observed that each 50 μg/m^3^ increment in PM_2.5_ resulted in a 1.943 mg/dl increment of HDL-C (*p*-value: 0.012) [39]. However, prolonged exposure to PM_2.5_ has been linked to higher levels of TC, TG, as well as LDL-C, and lower HDL-C [3,13,15]. For example, an increase of 10 μg/m^3^ in a three-year averaged PM_2.5_ level was associated with the changes of −2.03% (95% CI: −1.69%, −2.37%), 2.23% (1.44%, 3.02%), 0.92% (0.64%, 1.20%), and 3.04% (2.61%, 3.47%) in HDL-C, TG, TC, and LDL-C, respectively [13]. Additionally, a cross-sectional study conducted in North Carolina, USA, indicated the four lipids and PM_2.5_ were positively correlated. They observed that, for an increment of 1 μg/m^3^ in a one-year averaged PM_2.5_, TG, TC, LDL-C, as well as HDL-C, increased 3.29% (95% CI: 1.67%, 4.92%), 1.62% (1.13%, 2.11%), 1.70% (1.02%, 2.37%), and 0.61% (0.07%, 1.13%), respectively [40]. A panel study in North Carolina reported that, as PM_2.5_ increased for each 1μg/m^3^, the TG and TC changed by −0.63 (95% CI: −2.29, −1.02) and −0.06 (−0.49, 0.36), but the statistics did not show any significant differences [17]. Additionally, a cross-sectional observation with data from the MESA Air (the Multi-Ethnic Study of Atherosclerosis Air Pollution) study suggested that an increase of PM_2.5_ (5 μg/m^3^) was not correlated to the level of HDL cholesterol (−0.05 mg/dL; 95% CI: −0.82, 0.71), but correlated to decreased counts of HDL particle (−0.64 μmol/L; 95%CI: −1.01, −0.26) [41].

As shown above, our results on the association of PM_2.5_–lipids differ from several previous studies, and the reasons can be approximately explained by the following points. Firstly, the average concentration of PM_2.5_ during our research (38.34 ± 18.33 μg/m^3^) did not exceed the given standard of the Chinese National Ambient Air Quality Standard for a 24-h average, 75 μg/m^3^ [42]. The mean concentrations of the other studies were 76.97 μg/m^3^ [15], 41.93 μg/m^3^ [13], and 82.02 μg/m^3^ [3], respectively. To the best of our knowledge, the above observed negative effect of fine particulate matter on TC, TG, as well as LDL-C, and the positive effect on HDL-C may be considered as the result of hormesis [43]. The second possible reason was that the study populations were different. Our study focused on healthy adults aged 18 to 30, while Yeatts et al. recruited 12 adult asthmatics aged 21–50 for their panel study [17]. McGuinn et al. focused on elderly cardiac catheterization patients [40]. Additionally, the results may vary because of different study designs. For example, several studies adopted the cross-sectional design [3,13,40,41], while another study [17] and our study conducted a longitudinal panel design. Additionally, the biological mechanisms underlying the association between air pollution and blood lipids have not been fully revealed, but several hypotheses have been proposed by previous studies. Firstly, the systemic inflammation and oxidative stress induced by air pollutants may directly disrupt lipid metabolism, resulting in changes of lipid levels [12,44]. Additionally, systemic inflammation and oxidative stress are also associated with vascular inflammation and endothelial dysfunction, which may further damage multiple organs such as adipose tissue and the brain [15,45,46]. Air pollution may also affect lipid levels by altering the DNA methylation of specific genes associated with lipid metabolism [3,47].

According to the interaction analyses in this study, the PM_2.5_–lipids association may be modulated by the HOMA-IR, an indicator of insulin resistance. The four lipids were negatively associated with PM_2.5_, and the associations were greater in the lower HOMA-IR group. For instance, each increase of 10-μg/m^3^ in PM_2.5_ over lag 16–24 h was negatively related to TG (%change: −3.52%; 95% CI: −5.53%, −1.52%), TC (−1.98%; −2.84%, −1.11%), LDL-C (−2.27%; −3.44%, −1.09%), and HDL-C (−1.80%; −2.78%, −0.81%) in the group with the lower HOMA-IR. However, in the higher group, the changing percentages for the four corresponding lipids were −1.76% (95% CI: −2.96%, −0.54%), −0.87% (−1.39%, −0.35%), −1.35% (−2.05%, −0.64%), and −0.89% (−1.48%, −0.30%), respectively. It was proved that the participants with the lower HOMA-IR would be more susceptible to PM_2.5_ acute exposure. Interestingly, one cross-sectional study in Hebei, China, enrolled 8917 participants and suggested that high blood glucose levels may enhance the adverse effects of ambient air pollutants on lipids over long-term exposure. Specifically, the risk of air pollution on dyslipidemia increased with the increments of fasting blood glucose, and an adjusted odds ratio (95% CI) of 1.171 (1.162, 1.189) was found by the interaction between blood glucose and PM_2.5_. Overall, another study found that PM_2.5_ had a greater adverse effect on lipids in the abnormal glucose group, whereas our study found the lipid-lowering effect of PM_2.5_ was more pronounced in the lower HOMA group. This may suggest that the effect of PM_2.5_ on lipids observed in our main model was subject to a modification of insulin resistance. However, the biological explanation and potential mechanisms of the interaction between air pollutants and glucose remain unknown, and further research should be needed to address the issue.

The study still has some limitations. Firstly, lots of air pollutants, including gaseous pollutants and particulate matter, will actually have some impact on blood lipids [48,49]. Nevertheless, due to limited funds and apparatus, we only included PM_2.5_ in our study. Secondly, the participants of our study were healthy adults, who were less susceptible to particulate matter than the vulnerable population. However, we still observed the effects of PM_2.5_ on blood lipid levels among the subjects. Thirdly, the majority (80%) of the subjects were female students, which may lead to bias in the results, even though we have adjusted for gender as a covariate. Finally, compared to more accurate methods, such as intravenous glucose tolerance testing, the HOMA-IR may be biased in assessing insulin resistance levels [19]. However, those more accurate methods are more complicated and expensive, and the HOMA-IR has been applied to assess insulin resistance in numerous previous studies [19,25,50].

## 5. Conclusions

In conclusion, a panel of short-term PM_2.5_ exposure among healthy adults associated with lower TG, TC, and LDL-C levels and higher HDL-C at specific exposure time windows. The PM_2.5_–blood lipids association may be modified by insulin resistance. The hormesis effect may explain the findings that short-term PM_2.5_ exposure played a beneficial role on lipids in healthy adults and the positive effect could be stronger when they were at low HOMA-IR levels. The health impact of particulate-matter exposure on humans is beyond doubt, but more observation and further exploration are still needed.

## Figures and Tables

**Table 1:** Summary of basic characteristics of the 70 participants.

	n (%)	Mean ± SD	Median (Q1, Q3)
Gender, n (%)			
Male	14 (20.0%)		
Female	56 (80.0%)		
Age (years)		20.37 ± 1.59	
BMI (kg/m^2^)		21.50 ± 2.75	
blood lipids (mmol/L)			
TG			0.90 (0.73, 1.12)
TC			4.57 (4.09, 5.21)
LDL-C			1.98 (1.63, 2.35)
HDL-C			1.38 (1.23, 1.54)

Person visits N = 480. Abbreviations: SD, standard deviation; BMI, body mass index; Q1, 25th percentile; Q3, 75th percentile; TG, Triglycerides; TC, Total Cholesterol; LDL-C, Low Density Lipoprotein Cholesterol; HDL-C, High Density Lipoprotein Cholesterol.

**Table 2:** Association between per 10 μg/m^3^ increment in PM_2.5_ and blood lipid levels.

PM_2.5_ Lags	TG (95%CI)		TC (95%CI)		LDL-C (95%CI)		HDL-C (95%CI)	
	Crude	Adjusted	Crude	Adjusted	Crude	Adjusted	Crude	Adjusted
8–16 h	**−0.99 (−1.81, −0.16)**	−0.70 (−1.54, 0.15)	0.09 (−0.27, 0.45)	0.08 (−0.28, 0.45)	−0.41 (−0.89, 0.08)	−0.39 (−0.88, 0.10)	0.27 (−0.13, 0.67)	0.23 (−0.19, 0.64)
16–24 h	−0.76 (−1.64, 0.14)	−0.44 (−1.38, 0.50)	−0.01 (−0.40, 0.37)	−0.05 (−0.45, 0.36)	**−0.70 (−1.21, −0.19)**	**−0.66 (−1.20, −0.12)**	−0.15 (−0.58, 0.28)	−0.22 (−0.67, 0.24)
24–32 h	**−1.06 (−1.77, −0.35)**	**−0.91 (−1.63, −0.18)**	−0.30 (−0.61, 0.01)	**−0.33 (−0.64, −0.01)**	**−0.81 (−1.22, −0.40)**	**−0.83 (−1.25, −0.42)**	−0.15 (−0.50, 0.19)	−0.20 (−0.55, 0.15)
32–40 h	**−0.71 (−1.40, −0.03)**	−0.43 (−1.14, 0.29)	−0.14 (−0.44, 0.16)	−0.20 (−0.51, 0.11)	**−0.75 (−1.14, −0.35)**	**−0.82 (−1.23, −0.41)**	−0.23 (−0.56, 0.10)	−0.30 (−0.65, 0.04)
40–48 h	−0.83 (−1.83, 0.18)	−0.58 (−1.60, 0.46)	−0.25 (−0.68, 0.19)	−0.26 (−0.71, 0.18)	−0.56 (−1.14, 0.03)	−0.46 (−1.06, 0.14)	−0.02 (−0.50, 0.47)	0.02 (−0.48, 0.53)
48–56 h	−0.85 (−1.79, 0.10)	−0.97 (−1.97, 0.04)	0.05 (−0.36, 0.47)	0.23 (−0.20, 0.67)	−0.26 (−0.81, 0.29)	−0.06 (−0.65, 0.53)	0.28 (−0.18, 0.74)	0.31 (−0.18, 0.81)
56–64 h	0.02 (-0.83, 0.88)	0.22 (−0.63, 1.08)	0.22 (−0.15, 0.58)	0.32 (−0.05, 0.69)	−0.41 (−0.90, 0.08)	−0.30 (−0.80, 0.20)	**0.57 (0.16, 0.98)**	**0.62 (0.21, 1.04)**
64–72 h	−0.01 (−0.73, 0.71)	0.05 (−0.68, 0.78)	−0.07 (−0.38, 0.24)	0.01 (−0.30, 0.33)	−0.15 (−0.56, 0.27)	−0.12 (−0.54, 0.30)	**0.67 (0.33, 1.01)**	**0.67 (0.32, 1.02)**
0–8 h	−0.13 (−0.97, 0.72)	0.09 (−0.75, 0.94)	0.30 (−0.06, 0.66)	0.16 (−0.04, 0.54)	−0.44 (−0.93, 0.04)	−0.27 (−0.75, 0.22)	0.29 (−0.12, 0.69)	0.31 (−0.10, 0.72)
0–16 h	−0.72 (−1.67, 0.23)	−0.27 (−1.23, 0.70)	0.25 (−0.16, 0.66)	0.33 (−0.09, 0.74)	−0.55 (−1.09, 0.004)	−0.45 (−1.00, 0.11)	0.36 (−0.10, 0.82)	0.32 (−0.15, 0.79)
0–24 h	−0.84 (−1.82, 0.16)	−0.34 (−1.36, 0.69)	0.17 (−0.25, 0.60)	0.24 (−0.20, 0.69)	**−0.68 (−1.25, −0.11)**	−0.58 (−1.16, 0.01)	0.20 (−0.28, −0.68)	0.15 (−0.34, 0.65)
0–32 h	**−1.10 (−2.06, −0.12)**	−0.65 (−1.66, 0.36)	−0.01 (−043, 0.41)	0.02 (−0.41, 0.46)	**−0.87 (−1.43, −0.31)**	**−0.84 (−1.41, −0.26)**	0.07 (−0.40, 0.55)	0.00 (−0.50, 0.49)
0–40 h	**−1.07 (−1.99, −0.14)**	−0.62 (−1.59, 0.36)	−0.06 (−0.46, 0.35)	−0.05 (−0.47, 0.37)	**−0.92 (−1.45, −0.38)**	**−0.93 (−1.48, −0.37)**	−0.03 (−0.48, 0.42)	−0.13 (−0.60, 0.35)
0–48 h	**−1.14 (−2.12, −0.15)**	−0.66 (−1.69, 0.38)	−0.10 (−0.52, 0.33)	−0.09 (−0.54, 0.36)	**−0.95 (−1.51, −0.38)**	**−0.94 (−1.53, −0.35)**	−0.03 (−0.51, 0.45)	−0.12 (−0.63, 0.38)
0–56 h	**−1.25 (−2.29, −0.20)**	−0.80 (−1.88, 0.30)	−0.08 (−0.54, 0.37)	−0.04 (−0.51, 0.43)	**−0.97 (−1.57, −0.36)**	**−0.92 (−1.55, −0.29)**	0.02 (−0.49, 0.53)	−0.08 (−0.61, 0.46)
0–64 h	**−1.15 (−2.23, −0.07)**	−0.68 (−1.79, 0.45)	−0.03 (−0.50, 0.44)	0.04 (−0.44, 0.53)	**−0.98 (−1.60, −0.36)**	**−0.91 (−1.55, −0.26)**	0.13 (−0.39, 0.66)	0.07 (−0.48, 0.62)
0–72 h	−1.06 (−2.15, 0.04)	−0.54 (−1.67, 0.60)	−0.05 (−0.52, 0.43)	0.06 (−0.43, 0.55)	**−0.93 (−1.56, −0.30)**	**−0.84 (−1.49, −0.18)**	0.30 (−0.23, 0.83)	0.26 (−0.30, 0.81)

Values indicated the percentage change with 95% confidence interval (95% CI) in blood lipids for each 10 μg/m^3^ increase in PM_2.5_ with diverse time windows. The crude model was not adjusted for confounders. The adjusted model was adjusted for temperature, humidity, age, gender, BMI, the day of the week, exercise status, alcohol, and caffeine. Abbreviations: CI, confidence interval. Bold indicates *p* < 0.05.

## Data Availability

Data available on request due to restrictions e.g., privacy or ethical.

## Supplementary Materials

The following supporting information can be downloaded at: https://www.mdpi.com/article/10.3390/toxics10110663/s1, Table S1: Summary statistics of personal 72-h PM_2.5_ exposure levels (μg/m^3^); Table S2: Summary statistics of blood lipids during the whole study period (mmol/L); Table S3: The interaction effects of HOMA-IR and PM_2.5_ on blood lipids; Table S4: Sensitivity analyses of lagged PM_2.5_ exposure on blood lipids.
