# Supplementary material for: Association of Short-Term Exposure to PM2.5 with Blood Lipids and the Modification Effects of Insulin Resistance: A Panel Study in Wuhan
Source: Toxics. 2022 Nov 4;10(11):663. doi: 10.3390/toxics10110663 (PMC9698404; doi:10.3390/toxics10110663)
Supplement: Supplementary file 1 [file toxics-10-00663-s001.zip › toxics-1938583-supplementary.pdf]

Association of Short-Term Exposure to $PM_{2.5}$ with Blood Lipids and the Modification Effects of Insulin Resistance: A Panel Study in Wuhan

Jinhui Sun [1,2], Shouxin Peng [1,2], Zhaoyuan Li [1,2], Feifei Liu [1,2], Chuangxin Wu [1,2], Yuanan Lu [3,*], Hao Xiang [1,2,*]

[1] Department of Global Health, School of Public Health, Wuhan University, 115# Donghu Road, Wuhan 430071, China

[2] Global Health Institute, Wuhan University, 115# Donghu Road, Wuhan 430071, China

[3] Environmental Health Laboratory, Department of Public Health Sciences, University of Hawaii at Manoa, 1960 East West Rd, Biomed Bldg, D105, Honolulu, HI 96822, USA

* Corresponding authors:

Dr. Yuanan Lu, Email: yuanan@hawaii.edu

Dr. Hao Xiang, Email: xianghao@whu.edu.cn

Table S1. Summary statistics of personal 72-h $PM_{2.5}$ exposure levels (μg/m^3).

Visits	Mean	SD	Min	Median	Max	IQR
1	32.02	8.74	20.89	31.06	86.02	7.36
2	16.04	6.72	7.38	13.91	38.51	9.86
3	31.45	5.22	19.39	30.94	50.39	6.75
4	28.32	8.47	10.50	28.79	44.15	13.59
5	54.45	11.15	28.51	55.12	81.78	16.89
6	63.25	21.71	34.15	57.96	115.38	20.76
7	46.60	10.84	16.31	45.11	71.60	11.14
8	51.08	12.46	29.10	50.15	70.79	22.86
Total	38.34	18.33	7.38	34.88	115.38	23.56

Abbreviations: $PM_{2.5}$, particulate matter with aerodynamic diameter < 2.5μm; SD, standard deviation; IQR, interquartile range.

Table S2. Summary statistics of blood lipids during the whole study period (mmol/L).

Visits	n	TG		TC		LDL-C		HDL-C	
		Mean	SD	Mean	SD	Mean	SD	Mean	SD
1	69	1.10	0.47	4.89	0.91	2.02	0.60	1.40	0.27
2	70	1.01	0.48	4.71	1.08	2.10	0.62	1.42	0.28
3	68	1.02	0.52	4.74	0.87	2.14	0.57	1.43	0.28
4	70	1.01	0.40	4.67	0.90	2.04	0.58	1.42	0.31
5	68	0.96	0.44	4.79	1.00	1.99	0.60	1.43	0.34
6	47	1.01	0.55	4.91	1.17	2.08	0.67	1.42	0.33
7	49	0.95	0.37	4.32	0.88	1.85	0.51	1.43	0.59
8	39	0.95	0.43	4.68	1.04	2.05	0.68	1.47	0.34
Total	480	1.00	0.46	4.72	0.98	2.04	0.60	1.42	0.34

Abbreviations: TG, Triglycerides; TC, Total Cholesterol; LDL-C, Low Density Lipoprotein Cholesterol; HDL-C, High Density Lipoprotein Cholesterol; SD, standard deviation.

Table S3. The interaction effects of HOMA-IR and $PM_{2.5}$ on blood lipids.

	TG (95%CI)	TC (95%CI)	LDL-C (95%CI)	HDL-C (95%CI)
Lag 8–16h				
lower	−3.43 (−5.27, −1.59)	−1.69 (−2.48, −0,90)	−1.78 (−2.86, −0.69)	−1.11 (−2.02, −0.20)
higher	−1.83 (−2.90, −0.74)	−0.65 (−1.11, −0.18)	−0.96 (−1.59, −0.33)	−0.33 (−0.85, 0.21)
p	**0.002**	**<0.001**	**0.006**	**0.002**
Lag 16–24h				
lower	−3.52 (−5.53, −1.52)	−1.98 (−2.84, −1.11)	−2.27 (−3.44, −1.09)	−1.80 (−2.78, −0.81)
higher	−1.76 (−2.96, −0.54)	−0.87 (−1.39, −0.35)	−1.35 (−2.05, −0.64)	−0.89 (−1.48, −0.30)
p	**0.001**	**<0.001**	**0.003**	**0.001**
Lag 24–32h				
lower	−3.49 (−5.23, −1.76)	−2.18 (−2.92, −1.44)	−2.25 (−3.26, −1.23)	−1.96 (−2.80, −1.12)
higher	−1.98 (−2.96, −1.00)	−1.10 (−1.51, −0.68)	−1.42 (−1.99, −0.85)	−0.93 (−1.40, −0.46)
p	**0.002**	**<0.001**	**0.003**	**<0.001**
Lag 32–40h				
lower	−2.76 (−4.37, −1.16)	−1.86 (−2.55, −1.18)	−2.14 (−3.07, −1.20)	−1.92 (−2.68, −1.15)
higher	−1.38 (−2.30, −0.45)	−0.88 (−1.27, −0.49)	−1.36 (−1.89, −0.82)	−0.96 (−1.40, −0.52)
p	**0.002**	**<0.001**	**0.003**	**<0.001**
Lag 40–48h				
lower	−3.37 (−5.42, −1.31)	−1.92 (−2.81, −1.03)	−1.77 (−2.98, −0.55)	−1.75 (−2.75, −0.74)
higher	−1.69 (−2.93, −0.43)	−0.93 (−1.46, −0.39)	−0.98 (−1.71, −0.24)	−0.69 (−1.29, −0.07)
p	**0.003**	**<0.001**	**0.018**	**<0.001**
Lag 48-56h				
lower	−3.09 (−5.03, −1.15)	−0.87 (−1.72, −0.01)	−0.96 (−2.12, 0.21)	−0.89 (−1.84, 0.07)
higher	−1.72 (−2.88, −0.55)	−0.15 (−0.66, 0.36)	−0.38 (−1.07, 0.32)	−0.11 (−0.68, 0.46)
p	**0.015**	**0.004**	**0.084**	**0.005**
Lag 56–64h				
lower	−2.58 (−4.32, −0.83)	−0.91 (−1.67, −0.15)	−1.38 (−2.42, −0.34)	−0.75 (−1.6−, 0.11)
higher	−0.84 (−1.86, 0.20)	−0.14 (−0.59, 0.30)	−0.71 (−1.32, −0.10)	0.11 (−0.39, 0.61)
p	**0.001**	**<0.001**	**0.024**	**<0.001**
Lag 64–72h				
lower	−2.58 (−4.32, −0.83)	−1.00 (−1.74, −0.27)	−0.95 (−1.93, 0.05)	−0.39 (−1.20, 0.43)
higher	−0.92 (−1.85, 0.03)	−0.39 (−0.80, 0.02)	−0.45 (−1.00, 0.11)	0.25 (−0.20, 0.71)
p	**0.002**	**0.003**	**0.077**	**0.006**

Adjusted for ambient temperature, relative humidity, age, gender, BMI, the day of the week, exercise status, alcohol drink, and caffeine; bold indicates p-value for interaction < 0.05. Abbreviations: CI, confidence interval; $PM_{2.5}$, particulate matter with aerodynamic diameter < 2.5μm; TG, Triglycerides; TC, Total Cholesterol; LDL-C, Low Density Lipoprotein Cholesterol; HDL-C, High Density Lipoprotein Cholesterol; HOMA-IR, homeostasis model assessment of insulin resistance.

Table S4. Sensitivity analyses of lagged $PM_{2.5}$ exposure on blood lipids.

	TG (95%CI)	TC (95%CI)	LDL-C (95%CI)	HDL-C (95%CI)
Lag 8–16h				
Model 1	−0.62 (−1.47, 0.24)	0.08 (−0.29, 0.45)	−0.41 (−0.90, 0.09)	0.22 (−0.20, 0.64)
Model 2	−0.66 (−1.51, 0.20)	0.07 (−0.29, 0.44)	−0.40 (−0.89, 0.10)	0.22 (−0.19, 0.64)
Model 3	−0.66 (−1.51, 0.20)	0.04 (−0.32, 0.41)	−0.41 (−0.90, 0.08)	0.20 (−0.21, 0.61)
Lag 16–24h				
Model 1	−0.39 (−1.34, 0.57)	−0.04 (−0.45, 0.37)	**−0.60 (−1.15, −0.05)**	−0.21 (−0.68, 0.26)
Model 2	−0.40 (−1.34, 0.56)	−0.04 (−0.45, 0.37)	**−0.66 (−1.21, −0.11)**	−0.19 (−0.65, 0.27)
Model 3	−0.42 (−1.36, 0.54)	−0.04 (−0.44, 0.37)	**−0.62 (−1.17, −0.08)**	−0.25 (−0.71, 0.20)
Lag 24–32h				
Model 1	**−0.96 (−1.68, −0.22)**	**−0.31 (−0.65, −0.02)**	**−0.78 (−1.20, −0.36)**	−0.20 (−0.57, 0.16)
Model 2	**−0.89 (−1.62, −0.16)**	**−0.33 (−0.65, −0.01)**	**−0.84 (−1.26, −0.41)**	−0.19 (−0.55, 0.16)
Model 3	**−0.85 (−1.58, −0.12)**	**−0.35 (−0.66, −0.03)**	**−0.84 (−1.26, −0.42)**	−0.22 (−0.58, 0.13)
Lag 32–40h				
Model 1	−0.42 (−1.15, 0.31)	−0.20 (−0.51, 0.11)	**−0.79 (−1.20, −0.37)**	−0.32 (−0.67, 0.04)
Model 2	−0.44 (−1.16, 0.29)	−0.21 (−0.52, 0.11)	**−0.82 (−1.23, −0.40)**	−0.30 (−0.65, 0.05)
Model 3	−0.35 (−1.07, 0.38)	−0.24 (−0.55, 0.08)	**−0.84 (−1.26, −0.43)**	−0.34 (−0.69, 0.01)
Lag 40–48h				
Model 1	−0.54 (−1.58, 0.51)	−0.30 (−0.75, 0.15)	−0.50 (−1.10, 0.11)	0.01 (−0.50, 0.53)
Model 2	−0.57 (−1.61, 0.29)	−0.27 (−0.72, 0.19)	−0.45 (−1.06, 0.15)	0.02 (−0.49, 0.53)
Model 3	−0.43 (−1.48, 0.62)	−0.30 (−0.75, 0.15)	−0.47 (−1.07, 0.14)	−0.03 (−0.54, 0.48)
Lag 48–56h				
Model 1	−0.84 (−1.87, 0.18)	0.20 (−0.25, 0.64)	−0.05 (−0.65, 0.55)	0.31 (−0.19, 0.82)
Model 2	−1.02 (−2.03, 0.003)	0.24 (−0.20, 0.68)	−0.03 (−0.63, 0.57)	0.34 (−0.16, 0.83)
Model 3	−0.91 (−1.92, 0.10)	0.22 (−0.21, 0.66)	−0.04 (−0.63, 0.55)	0.28 (−0.21, 0.78)
Lag 56–64h				
Model 1	0.18 (−0.68, 1.05)	0.32 (−0.05, 0.70)	−0.26 (−0.76, 0.24)	**0.64 (0.22, 1.06)**
Model 2	0.19 (−0.67, 1.06)	0.33 (−0.04, 0.71)	−0.28 (−0.78, 0.22)	**0.63 (0.22, 1.05)**
Model 3	0.34 (−0.53, 1.21)	0.30 (−0.06, 0.68)	−0.31 (−0.81, 0.19)	**0.61 (0.19, 1.03)**
Lag 64–72h				
Model 1	0.0003 (−0.73, 0.74)	0.01 (−0.30, 0.33)	−0.09 (−0.52, 0.33)	**0.70 (0.35, 1.06)**
Model 2	0.03 (−0.70, 0.77)	0.02 (−0.30, 0.34)	−0.09 (−0.52, 0.33)	**0.70 (0.35, 1.05)**
Model 3	0.09 (−0.64, 0.82)	0.03 (−0.29, 0.34)	−0.10 (−0.52, 0.33)	**0.66 (0.31, 1.01)**

Model 1, only including those students who completed more than 5 visits; Model 2, excluding those whose CRP was over 10 mg/L; Model 1 and Model 2 both adjusting for ambient temperature, relative humidity, age, gender, BMI, the day of the week, exercise status, alcohol drink, and caffeine; Model 3 further adjusting for diet included fat, fish, egg and fried food consumption; bold indicates $p < 0.05$. Abbreviations: CI, confidence interval; $PM_{2.5}$, particulate matter with aerodynamic diameter < 2.5μm; TG, Triglycerides; TC, Total Cholesterol; LDL-C, Low Density Lipoprotein Cholesterol; HDL-C, High Density Lipoprotein Cholesterol.
